# *N*-(Naphthalen-1-yl­methyl­idene)-4*H*-1,2,4-triazol-4-amine

**DOI:** 10.1107/S1600536812039104

**Published:** 2012-09-22

**Authors:** Pan Yang, Bin Ding, Gui-Xiang Du

**Affiliations:** a Tianjin Key Laboratory of Structure and Performance for Functional Molecules, Tianjin Normal University, Tianjin 300071, People’s Republic of China

## Abstract

In the title mol­ecule, C_13_H_10_N_4_, the dihedral angle between the triazole ring and the naphthalene ring system is is 56.1 (2)°. In the crystal, mol­ecules are connected by weak C—H⋯N hydrogen bonds into chains along [100]. A short intra­molecular C—H⋯N contact is also observed.

## Related literature

For applications of triazole derivatives, see: Demirbas *et al.* (2002 ▶); Foroumadi *et al.* (2003 ▶); He *et al.* (2006 ▶); Kritsanida *et al.* (2002 ▶); Manfredini *et al.* (2000 ▶). For standard bond lengths, see: Allen *et al.* (1987 ▶).
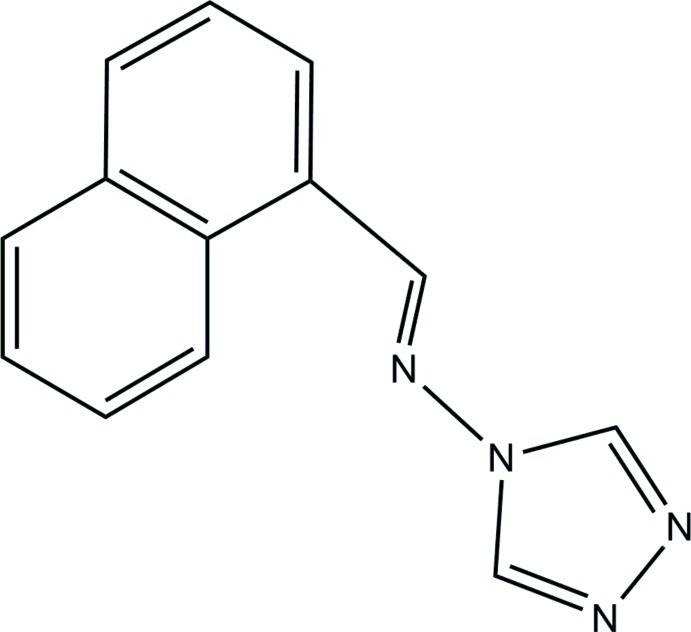


## Experimental

### 

#### Crystal data

C_13_H_10_N_4_
*M* *_r_* = 222.25
Monoclinic, 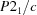

*a* = 5.499 (3) Å
*b* = 10.079 (6) Å
*c* = 19.942 (12) Å
β = 92.758 (7)°
*V* = 1104.0 (11) Å^3^
*Z* = 4
Mo *K*α radiation
μ = 0.09 mm^−1^
*T* = 296 K
0.2 × 0.15 × 0.1 mm

#### Data collection

Bruker APEXII CCD diffractometer
Absorption correction: multi-scan (*SADABS*; Sheldrick, 1996 ▶) *T*_min_ = 0.5, *T*_max_ = 1.0
6574 measured reflections
2168 independent reflections
1787 reflections with *I* > 2σ(*I*)
*R*_int_ = 0.019

#### Refinement

*R*[*F* ^2^ > 2σ(*F* ^2^)] = 0.042
*wR*(*F* ^2^) = 0.113
*S* = 1.08
2168 reflections
154 parameters
H-atom parameters constrained
Δρ_max_ = 0.15 e Å^−3^
Δρ_min_ = −0.25 e Å^−3^

### 

Data collection: *APEX2* (Bruker, 2008 ▶); cell refinement: *SAINT* (Bruker, 2008 ▶); data reduction: *SAINT*; program(s) used to solve structure: *SHELXS97* (Sheldrick, 2008 ▶); program(s) used to refine structure: *SHELXL97* (Sheldrick, 2008 ▶); molecular graphics: *SHELXTL* (Sheldrick, 2008 ▶); software used to prepare material for publication: *publCIF* (Westrip, 2010 ▶).

## Supplementary Material

Crystal structure: contains datablock(s) global, I. DOI: 10.1107/S1600536812039104/lh5529sup1.cif

Structure factors: contains datablock(s) I. DOI: 10.1107/S1600536812039104/lh5529Isup2.hkl

Supplementary material file. DOI: 10.1107/S1600536812039104/lh5529Isup3.cml

Additional supplementary materials:  crystallographic information; 3D view; checkCIF report

## Figures and Tables

**Table 1:** Hydrogen-bond geometry (Å, °)

*D*—H⋯*A*	*D*—H	H⋯*A*	*D*⋯*A*	*D*—H⋯*A*
C9—H9*A*⋯N1	0.93	2.36	2.914 (2)	118
C13—H13*A*⋯N4^i^	0.93	2.45	3.330 (3)	157

Symmetry code: (i) 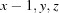.

**Fig. 1.**
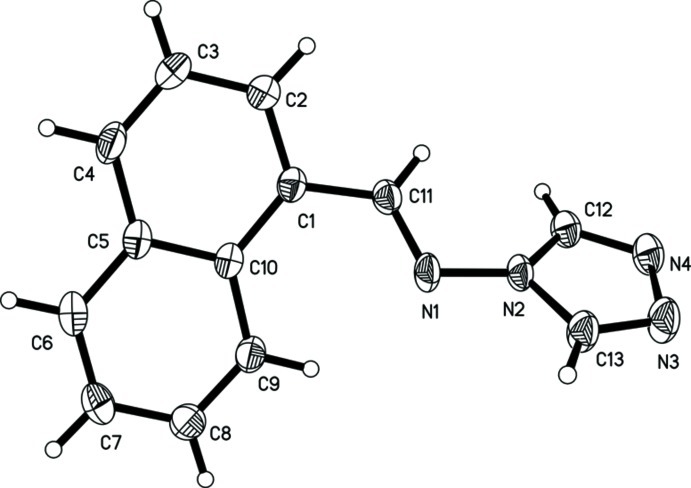
The molecular structure of the title compound. Displacement ellipsoids are drawn at the 30% probability level.

**Fig. 2.**
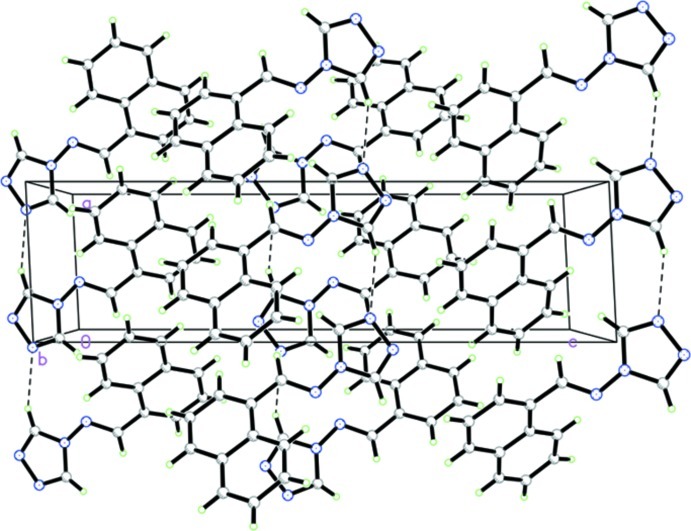
Part of the crystal structure of the title compound, showing weak hydrogen bonds as dashed lines.

## supplementary crystallographic information

### Comment

1,2,4-Triazole is a basic aromatic ring and possesses good coordination ability due to the presence of nitrogen atoms. 1,2,4-Triazole derivatives can be used to build polymetallic complexes (He *et al.*, 2006). Compounds derived from triazole possess antimicrobial, analgesic, anti-inflammatory, local anesthetic, antineoplastic and antimalarial properties (Foroumadi *et al.*, 2003). Some triazole Schiff bases also exhibit antiproliferative and anticancer activities (Manfredini *et al.*, 2000). Due to their significant biological applications, triazoles have gained much attention in bioinorganic and metal-based drug discovery (Demirbas *et al.*, 2002; Kritsanida *et al.*, 2002).

The molecular structure of the title compound is shown in Fig. 1. The dihedral angle between the triazole and naphthalene ring system is is 56.1 (2)°. The C—N and C═N bond lengths agree with standard values (Allen *et al.*, (1987) and show the obvious effects of electron delocalization. In the crystal, molecules are connected by weak C—H···N hydrogen bonds into chains along [100] (Fig. 2).

### Experimental

A mixture of 1-naphthaldehyde (10 mmol) and 4-amino-4H-1,2,4-triazole (10 mmol) in ethanol (20 mL) was refluxed on a steam-bath for 30 min. The colour of the solution changed to reddish-orange and was kept under ice-cold conditions to obtain a white solid product. Single crystals were formed in the mother liquor after ten days.

### Refinement

H atoms were positioned geometrically (C-H = 0.93 Å) and allowed to ride on their parent atoms, with U_iso_(H) = 1.2 U_eq_(C).

### Crystal data

**Table:**

C_13_H_10_N_4_	*F*(000) = 464
*M**_r_* = 222.25	*D*_x_ = 1.337 Mg m^−^^3^
Monoclinic, *P*2_1_/*c*	Mo *K*α radiation, λ = 0.71073 Å
Hall symbol: -P 2ybc	Cell parameters from 6574 reflections
*a* = 5.499 (3) Å	θ = 2.0–26°
*b* = 10.079 (6) Å	µ = 0.09 mm^−^^1^
*c* = 19.942 (12) Å	*T* = 296 K
β = 92.758 (7)°	Block, colorless
*V* = 1104.0 (11) Å^3^	0.2 × 0.15 × 0.1 mm
*Z* = 4	

### Data collection

**Table:**

Bruker APEXII CCD diffractometer	2168 independent reflections
Radiation source: fine-focus sealed tube	1787 reflections with *I* > 2σ(*I*)
Graphite monochromator	*R*_int_ = 0.019
φ and ω scans	θ_max_ = 26.0°, θ_min_ = 2.0°
Absorption correction: multi-scan (*SADABS*; Sheldrick, 1996)	*h* = −6→6
*T*_min_ = 0.5, *T*_max_ = 1.0	*k* = −12→12
6574 measured reflections	*l* = −24→24

### Refinement

**Table:**

Refinement on *F*^2^	0 restraints
Least-squares matrix: full	H-atom parameters constrained
*R*[*F*^2^ > 2σ(*F*^2^)] = 0.042	*w* = 1/[σ^2^(*F*_o_^2^) + (0.0598*P*)^2^ + 0.1522*P*] where *P* = (*F*_o_^2^ + 2*F*_c_^2^)/3
*wR*(*F*^2^) = 0.113	(Δ/σ)_max_ = 0.001
*S* = 1.08	Δρ_max_ = 0.15 e Å^−^^3^
2168 reflections	Δρ_min_ = −0.25 e Å^−^^3^
154 parameters	

### Special details

**Table:**

Geometry. All e.s.d.'s (except the e.s.d. in the dihedral angle between two l.s. planes) are estimated using the full covariance matrix. The cell e.s.d.'s are taken into account individually in the estimation of e.s.d.'s in distances, angles and torsion angles; correlations between e.s.d.'s in cell parameters are only used when they are defined by crystal symmetry. An approximate (isotropic) treatment of cell e.s.d.'s is used for estimating e.s.d.'s involving l.s. planes.
Refinement. Refinement of *F*^2^ against ALL reflections. The weighted *R*-factor *wR* and goodness of fit *S* are based on *F*^2^, conventional *R*-factors *R* are based on *F*, with *F* set to zero for negative *F*^2^. The threshold expression of *F*^2^ > σ(*F*^2^) is used only for calculating *R*-factors(gt) *etc*. and is not relevant to the choice of reflections for refinement. *R*-factors based on *F*^2^ are statistically about twice as large as those based on *F*, and *R*- factors based on ALL data will be even larger.

### Fractional atomic coordinates and isotropic or equivalent isotropic displacement parameters (Å^2^)

**Table:**

	*x*	*y*	*z*	*U*_iso_*/*U*_eq_	
N1	0.6439 (2)	0.79790 (12)	0.97839 (6)	0.0429 (3)	
N2	0.8048 (2)	0.84041 (11)	1.02686 (6)	0.0384 (3)	
N3	0.9117 (2)	0.91095 (15)	1.12480 (6)	0.0570 (4)	
N4	1.1212 (2)	0.88352 (14)	1.08514 (6)	0.0517 (3)	
C1	0.5720 (2)	0.77204 (14)	0.86143 (6)	0.0382 (3)	
C2	0.6174 (3)	0.84224 (16)	0.80284 (7)	0.0494 (4)	
H2A	0.7474	0.9012	0.8024	0.059*	
C3	0.4736 (3)	0.82456 (17)	0.74739 (8)	0.0595 (5)	
H3A	0.5001	0.8713	0.7082	0.071*	
C4	0.2871 (3)	0.73591 (17)	0.75027 (7)	0.0544 (4)	
H4A	0.1853	0.7253	0.7120	0.065*	
C5	0.2370 (3)	0.65767 (13)	0.80837 (7)	0.0400 (3)	
C6	0.0469 (3)	0.56297 (15)	0.81066 (8)	0.0489 (4)	
H6A	−0.0576	0.5540	0.7729	0.059*	
C7	0.0069 (3)	0.48337 (16)	0.86520 (8)	0.0539 (4)	
H7A	−0.1223	0.4238	0.8643	0.065*	
C8	0.1586 (3)	0.49371 (15)	0.91971 (8)	0.0518 (4)	
H8A	0.1405	0.4386	0.9565	0.062*	
C9	0.3399 (3)	0.58640 (14)	0.92032 (7)	0.0445 (4)	
H9A	0.4410	0.5938	0.9589	0.053*	
C10	0.3841 (2)	0.67329 (13)	0.86498 (6)	0.0356 (3)	
C11	0.7224 (3)	0.80772 (13)	0.91754 (7)	0.0398 (3)	
H11A	0.8792	0.8386	0.9114	0.048*	
C12	1.0518 (3)	0.84233 (14)	1.02727 (7)	0.0437 (4)	
H12A	1.1494	0.8185	0.9924	0.052*	
C13	0.7272 (3)	0.88263 (17)	1.08876 (7)	0.0489 (4)	
H13A	0.5666	0.8887	1.1011	0.059*	

### Atomic displacement parameters (Å^2^)

**Table:**

	*U*^11^	*U*^22^	*U*^33^	*U*^12^	*U*^13^	*U*^23^
N1	0.0343 (6)	0.0541 (7)	0.0390 (6)	−0.0046 (5)	−0.0129 (5)	−0.0057 (5)
N2	0.0317 (6)	0.0439 (6)	0.0385 (6)	−0.0033 (5)	−0.0114 (5)	−0.0025 (5)
N3	0.0428 (8)	0.0794 (10)	0.0471 (7)	−0.0031 (7)	−0.0143 (6)	−0.0101 (7)
N4	0.0383 (7)	0.0617 (8)	0.0534 (7)	−0.0040 (6)	−0.0154 (6)	−0.0039 (6)
C1	0.0372 (7)	0.0403 (7)	0.0362 (7)	0.0026 (6)	−0.0062 (5)	−0.0008 (5)
C2	0.0502 (9)	0.0541 (9)	0.0432 (8)	−0.0083 (7)	−0.0050 (7)	0.0064 (6)
C3	0.0709 (11)	0.0685 (11)	0.0378 (8)	−0.0079 (9)	−0.0108 (7)	0.0141 (7)
C4	0.0592 (10)	0.0630 (10)	0.0389 (8)	0.0006 (8)	−0.0188 (7)	0.0019 (7)
C5	0.0385 (8)	0.0413 (7)	0.0393 (7)	0.0056 (6)	−0.0082 (6)	−0.0065 (6)
C6	0.0434 (8)	0.0501 (9)	0.0516 (8)	0.0021 (7)	−0.0130 (7)	−0.0127 (7)
C7	0.0499 (9)	0.0480 (9)	0.0631 (10)	−0.0109 (7)	−0.0042 (7)	−0.0123 (7)
C8	0.0653 (10)	0.0427 (8)	0.0471 (8)	−0.0082 (7)	−0.0005 (7)	0.0007 (6)
C9	0.0536 (9)	0.0406 (8)	0.0384 (7)	−0.0030 (7)	−0.0088 (6)	−0.0012 (6)
C10	0.0364 (7)	0.0361 (7)	0.0337 (7)	0.0052 (5)	−0.0045 (5)	−0.0046 (5)
C11	0.0350 (7)	0.0406 (7)	0.0429 (8)	−0.0030 (6)	−0.0078 (6)	0.0002 (6)
C12	0.0337 (7)	0.0489 (8)	0.0475 (8)	−0.0005 (6)	−0.0079 (6)	−0.0018 (6)
C13	0.0356 (8)	0.0692 (10)	0.0411 (8)	−0.0031 (7)	−0.0073 (6)	−0.0067 (7)

### Geometric parameters (Å, º)

**Table:**

N1—C11	1.311 (2)	C4—H4A	0.9300
N1—N2	1.3486 (16)	C5—C10	1.3661 (19)
N2—C12	1.3577 (19)	C5—C6	1.418 (2)
N2—C13	1.392 (2)	C6—C7	1.378 (2)
N3—C13	1.2481 (19)	C6—H6A	0.9300
N3—N4	1.455 (2)	C7—C8	1.342 (2)
N4—C12	1.2679 (19)	C7—H7A	0.9300
C1—C2	1.399 (2)	C8—C9	1.366 (2)
C1—C11	1.4059 (19)	C8—H8A	0.9300
C1—C10	1.438 (2)	C9—C10	1.439 (2)
C2—C3	1.340 (2)	C9—H9A	0.9300
C2—H2A	0.9300	C11—H11A	0.9300
C3—C4	1.363 (2)	C12—H12A	0.9300
C3—H3A	0.9300	C13—H13A	0.9300
C4—C5	1.439 (2)		
			
C11—N1—N2	113.90 (12)	C5—C6—H6A	117.9
N1—N2—C12	129.10 (12)	C8—C7—C6	118.53 (15)
N1—N2—C13	120.92 (12)	C8—C7—H7A	120.7
C12—N2—C13	109.85 (11)	C6—C7—H7A	120.7
C13—N3—N4	106.65 (13)	C7—C8—C9	119.02 (15)
C12—N4—N3	110.20 (12)	C7—C8—H8A	120.5
C2—C1—C11	114.43 (13)	C9—C8—H8A	120.5
C2—C1—C10	123.27 (12)	C8—C9—C10	124.04 (13)
C11—C1—C10	122.29 (12)	C8—C9—H9A	118.0
C3—C2—C1	120.01 (15)	C10—C9—H9A	118.0
C3—C2—H2A	120.0	C5—C10—C9	116.64 (13)
C1—C2—H2A	120.0	C5—C10—C1	115.93 (12)
C2—C3—C4	117.87 (15)	C9—C10—C1	127.39 (12)
C2—C3—H3A	121.1	N1—C11—C1	120.65 (13)
C4—C3—H3A	121.1	N1—C11—H11A	119.7
C3—C4—C5	124.52 (13)	C1—C11—H11A	119.7
C3—C4—H4A	117.7	N4—C12—N2	105.52 (13)
C5—C4—H4A	117.7	N4—C12—H12A	127.2
C10—C5—C6	117.34 (13)	N2—C12—H12A	127.2
C10—C5—C4	118.24 (14)	N3—C13—N2	107.76 (14)
C6—C5—C4	124.40 (13)	N3—C13—H13A	126.1
C7—C6—C5	124.29 (13)	N2—C13—H13A	126.1
C7—C6—H6A	117.9		

### Hydrogen-bond geometry (Å, º)

**Table:**

*D*—H···*A*	*D*—H	H···*A*	*D*···*A*	*D*—H···*A*
C9—H9*A*···N1	0.93	2.36	2.914 (2)	118
C13—H13*A*···N4^i^	0.93	2.45	3.330 (3)	157

Symmetry code: (i) *x*−1, *y*, *z*.
